# End-stage knee osteoarthritis with and without sarcopenia and the effect of knee arthroplasty – a prospective cohort study

**DOI:** 10.1186/s12877-020-01929-6

**Published:** 2021-01-04

**Authors:** Kevin Ki-Wai Ho, Lawrence Chun-Man Lau, Wai-Wang Chau, Queena Poon, Kwong-Yin Chung, Ronald Man-Yeung Wong

**Affiliations:** 1 Department of Orthopaedics and Traumatology, Chinese University of Hong Kong, Shatin, Hong Kong SAR, China; 2 Department of Orthopaedics and Traumatology, Prince of Wales Hospital, Shatin, Hong Kong SAR, China

**Keywords:** Knee arthroplasty, Sarcopenia, Knee osteoarthritis, Sarcopenic obesity, Cohort study

## Abstract

**Background:**

Sarcopenia often accompanies osteoarthritis (OA), which is managed by total knee arthroplasty (TKA) in the late stage. Recent studies have suggested a higher risk of post-operative complications after TKA in sarcopenic OA subjects, but whether TKA can benefit them similar to non-sarcopenic subjects remains unexplored. This study aimed to examine the dynamic, mutual impact of sarcopenia and TKA in a one-year post-operative period.

**Methods:**

This prospective cohort study was conducted between 2015 to 2018 at our hospital. Patients with end-stage OA of the knee waiting for TKA were recruited into the study. Primary outcome measures were change in muscle strength, mass and function. Secondary outcome measures were quality of life (QOL) measurements for pain, psychological and physical health.

**Results:**

Fifty-eight patients were recruited, of which 79.3% were female and 32.8% already had sarcopenia at baseline. The average age of sarcopenic subjects and non-sarcopenic subjects was comparable (67.89 ± 7.07 vs. 67.92 ± 6.85; *p* = 0.99), but sarcopenic subjects had a lower body mass index (BMI) (25.64 ± 2.64 vs. 28.57 ± 4.04; *p* = 0.01). There was a statistically significant improvement in walking speed (10.24 ± 5.35 vs. 7.69 ± 2.68, *p* < 0.01) and muscle strength in both sarcopenic and non-sarcopenic patients after TKA. This was accompanied by an improvement trend in muscle mass in all subjects. There was no change in handgrip power before and after TKA and subsequent follow-up (19.31 ± 5.92 vs. 18.98 ± 6.37 vs. 19.36 ± 7.66; *p* = 0.97). QOL measured before, after and at follow-up with WOMAC (total: 42.27 ± 15.98 vs. 20.65 ± 15.24 vs. 16.65 ± 18.13) and SF12v2 (PCS: 33.06 ± 8.55 vs. 38.96 ± 8.01 vs. 40.67 ± 7.93) revealed progressive significant improvement (both comparisons *p* ≤ 0.01). Further analysis with the IPAQ also found increased engagement of high-intensity activities.

**Conclusions:**

This study showed that sarcopenia among patients with end-stage OA of the knee is not uncommon, but both sarcopenic and non-sarcopenic OA patients achieved significant clinical and functional improvement after TKA. Further studies with a larger sample size and different ethnicities could help ascertain a beneficial role of TKA in sarcopenic OA subjects.

**Trial registration:**

Registry: ClinicalTrials.gov, Registration number: NCT03579329. Date of registration: 6 July 2018. Retrospectively registered.

## Background

Sarcopenia, defined as age-related decline in muscle mass and strength, is a common condition in the aging population resulting in significant functional impairment and inactivity [1–3]. The prevalence of sarcopenia increases with age, reaching an astounding 50% among the population aged 75 or above in the United States [4]. Sarcopenia is often associated with frailty, falls, fractures and disability in this susceptible population [5–7]. Furthermore, the disease is a strong predictive risk factor for mortality and morbidity among older adult patients in nursing homes [8].

Notably, sarcopenia often accompanies osteoarthritis (OA). However, the relationship between sarcopenia and OA is still unclear and no strong consensus has been reached [9, 10]. Sarcopenia and OA have been postulated to be co-existing conditions [11, 12]’ conversely, sarcopenia may be a risk factor for OA progression [13] and vice versa, with an increased risk of sarcopenia in patients with OA [14]. Cross-sectional studies have revealed that OA in the knee is associated with declines in muscle mass and strength in the lower limbs as the patient adapts to a sedentary lifestyle and inactivity to avoid knee joint pain and stiffness [15–18]. In turn, the subsequent reduction in energy expenditure, together with ageing-related gains in adipose tissue, lead to these patients to develop overweight or even obesity. This increased load further exacerbates knee OA progression, and it is the combination of these factors that is considered to create and perpetuate a vicious cycle between obesity, sarcopenia and osteoarthritis [19, 20].

Patients with end-stage OA of the knee will eventually pursue total knee arthroplasty (TKA) as the only viable option. TKA has been proven to relieve pain and regain mobility. It has been widely accepted that TKA greatly increases social and physical aspects of quality of life [21–24]. Despite the common coprevalence of sarcopenia and OA, reports on the impact of sarcopenia on end-stage OA patients undergoing TKA are limited to two recent retrospective case-control studies, suggesting that patients with sarcopenia undergoing primary TKA have greater in-hospital length of stay, increased odds of 90-day medical complications, falls, lower extremity fractures, prosthetic joint infection and reoperations [25, 26]. Frailty, a condition closely associated with sarcopenia, has also been linked to a higher rate of mortality, post-operative admission to the intensive care unit, discharge to institutional care and re-admission in a recent population-based study of patients undergoing TKA [27]. However, whether TKA can benefit sarcopenic OA subjects similar to non-sarcopenic OA subjects is currently unexplored. It is not known, after TKA-related improvements in knee symptoms, whether sarcopenic OA subjects can attain significant improvements in muscle strength, muscle mass and gait speed, which are the main domains that define sarcopenia. It may be possible that, after TKA, sarcopenic patients can participate in more activities to improve their muscle strength and gait speed, as they are free from knee pain. A longitudinal study that examines and observes changes in sarcopenic features after TKA over time can bridge the knowledge gap in this aspect and can provide insight into how to best manage patients with concomitant OA and sarcopenia.

This study aimed to examine the status of sarcopenia in individuals with symptomatic end-stage OA of the knee and the subsequent interaction between sarcopenia and TKA, which was employed as a definitive treatment for OA. It was hypothesised that, after TKA, sarcopenic patients would have improved knee symptoms and function, similar to non-sarcopenic patients.

## Methods

This study was conducted in compliance with the Declaration of Helsinki and was approved by The Joint Chinese University of Hong Kong – New Territories East Cluster Clinical Research Ethics Committee (Ethics approval number: 2015.539). The study was retrospectively registered in July 2018 with US ClinicalTrials.gov (Registration number: NCT03579329).

This prospective study was conducted at the Prince of Wales Hospital, Hong Kong from 1st November 2015 to 30th May 2018. Consecutive patients visiting the Orthopaedics Specialist Outpatient Clinic with symptomatic end-stage OA of the knee referred and opted for TKA as treatment were invited to participate in the study. Radiographic severity of knee OA was assessed and documented based on the Kellgren and Lawrence classification [28]. Clinical diagnosis of knee OA was based on medical history and clinical examination of knee joints. Clinical diagnosis of sarcopenia was examined using the Asian Working Group for Sarcopenia (AWGS) algorithm after they are recruited into the study [29].

### Sample size

The estimated study sample size is 50. Sample size was calculated using G*Power 3.1.9. This calculation was based upon DXA parameter being an indicator of sarcopenia [30]. As there are no similar previous studies, the sample size was calculated based on our pilot data of the present study comparing the DXA data measured at recruitment and after 12 months. Results showed DXA difference increased from the mean values of 5.84 to 6.02 after 12 months. Accounting for the 3.1% increase with the significant levels at 0.05 and power of 0.8 yielded a sample size of 45. Expecting a 10% withdrawal rate, a total of 50 subjects were required. Instead, researchers were able to finalise the recruitment of 58 end-stage OA knee patients upon their fulfilment of study prerequisites for this research.

### Eligibility criteria

The inclusion criteria were: (1) aged over 50 years with end-stage knee OA; (2) scheduled for TKA; (3) agreed to provide written consent and able to comply with study assessments. Exclusion criteria were: (1) history of connective tissue disorders or myositis; (2) previous period of alcoholism or drug abuse; (3) breastfeeding or pregnant women; (4) presence of serious pathologies with steroid-based systematic therapy in progress or interrupted for less than 1 month, or significant haematological disease; and (5) presence of significant cognitive impairment. The sarcopenia status was assessed by the AWGS algorithm after participant enrolment into the study.

### Physical measurements

Patient demographic data were recorded upon enrolment. Body weight and height were measured using a standard stadiometer and the body mass index (BMI) was calculated (bodyweight in kg/[height in m]^2^). Body composition at baseline and follow-up was measured using dual-energy X-ray absorptiometry (DXA) (Horizon, Hologic, Bedford, MA). Total appendicular skeletal muscle mass (ASM) was calculated by the sum of lean mass measured in the four limbs, with the operator adjusting the cut lines of the limbs as described by Heymsfield et al. [31] Knee flexion/extension muscle strength were measured by instructing the patient to perform an active knee flexion/extension movement in a sitting position with both feet off the ground, and the hip flexed at 90° and the knee joint in the mid-flexion range. The optimal isometric force of the knee flexion/extension movement was measured by a dynamometer attached at the malleoli level with a strap. The measurements were taken at maximum force for three times. Grip strength was measured as the mean values of 3 repeated and consecutive grip measurements on a dynamometer using the dominant hand. The six-meter gait speed test was used to measure the mean walking speed (in seconds) using after 3 attempts of walking for 6 m along a straight line.

### Definition of sarcopenia

Sarcopenia was defined by following the Asian Working Group for Sarcopenia (AWGS) algorithm [29]. A patient was told to be “sarcopenic” was based on multiple underlying outcomes. A person who has low muscle mass, low muscle strength and/or low physical performance was categorised as having sarcopenia. Low muscle mass was defined as height-adjusted muscle mass by DXA < 7.0 kg/m^2^ for men and < 5.4 kg/m^2^ for women; low muscle strength was defined as grip strength < 28 kg for men and < 18 kg for women; and low physical performance as gait speed < 1.0 m/s for both men and women.

### Outcomes

The primary outcome was DXA measurements. DXA values were used to produce the lean mass index (LMI), which is the ratio of total lean mass (soft tissue only, excluding bone) to height squared, and the appendage lean mass index (ALMI), which is the ratio of lean mass on the limbs to height squared.

Several measurements formed the secondary outcomes and assessments were consecutively conducted within 1 month before TKA (baseline), 6 months (post-treatment), and 12 months postoperatively. Quality of life (QOL) measurements were measured in terms of psychological and physical health. Pain, stiffness and physical functions of the Western Ontario and McMaster Universities Osteoarthritis Index (WOMAC) indicated scores ranging between 0 and 100, with higher scores being greater associable disability functions. Medical Outcomes Study Short Form 12 Health Survey Version 2 (SF-12v2) was administered quantify the general health status in subjects as a means to compute a physical and mental health composite score (PCS & MCS) ranging from zero to 100; a higher score indicates a better level of health. The International Physical Activity Questionnaire (IPAQ) is an internationally comparable record of health-related physical activity, used to monitor changes in the amount or type of exercise performance level over the research period. Physical activity levels in terms of IPAQ were categorised as “low”, “moderate” and “high” and the categorisation followed the standard criteria [32, 33]. The contraposition of SF-12v2 and IPAQ indexes across research timelines allowed for a meaningful interpretation of bodily and psychological functional fluctuations to assess the effect of TKA on sarcopenia symptoms. Handgrip (handgrip dynamometer at upper extremity strength), lower limb muscle strength in terms of knee joint flexion/extension and the 6-m gait speed test for lower extremity function were also recorded at the three time points.

### Statistical analysis

Demographic statistics on age, sex, BMI and length of hospital stay are reported in terms of mean ± SD or frequencies where appropriate (Table 1). Comparisons of ALMI and LMI against patients with or without sarcopenia were carried out both cross-sectionally (between patients with sarcopenia or not) and longitudinally (among the three time points, i.e. baseline, 6 months and 12 months) correspondingly. Longitudinal comparisons of mean values of PCS and MCS in SF12v2, WOMAC domain scores, IPAQ findings in terms of low, moderate, and high activities, knee flexion/extension strength, as well as handgrip scores and 6-m gait speed were made. To control for possible confounders, further longitudinal comparisons were performed by controlling sex, age and BMI using one-way ANOVA. *post-hoc* Bonferroni correction comparisons were carried out and presented using the log-rank test. A two-sided *p*-value ≤0.05 was considered statistically significant. All statistical analyses were carried out using IBM SPSS Version 26.0 (Armonk, NY: IBM Corp).

**Table 1:** Demographic characteristics of patients with or without sarcopenia (*N* = 58)

Demographic variables	Sarcopenia		*P* value
	Yes (*N* = 19)	No (*N* = 39)	
Age			
≤ 75	16 (84.2)	33 (84.6)	1.00
> 75	3 (15.8)	6 (15.4)	
Sex			
Male	5 (26.3)	7 (17.9)	0.50
Female	14 (73.7)	32 (82.1)	
BMI			
Normal	9 (47.4)	7 (17.9)	0.03
Overweight or obese	10 (52.6)	32 (82.1)	
Smoking			
Yes	0	0	–
No	19 (100.0)	39 (100.0)	
Drinking Behaviour			
Yes	0	0	–
No	19 (100.0)	39 (100.0)	
Diabetes Mellitus			
Yes	5 (26.3)	18 (46.2)	0.17
No	14 (73.7)	21 (53.8)	
Hypertension			
Yes	12 (63.2)	28 (71.8)	0.55
No	7 (36.8)	11 (28.2)	
Hyperlipidemia			
Yes	3 (15.8)	15 (38.5)	0.13
No	16 (84.2)	24 (61.5)	
Neurological disease			
Yes	0	3 (7.7)	0.54
No	19 (100.0)	36 (92.3)	
Renal disease			
Yes	2 (10.5)	1 (2.6)	0.25
No	17 (89.5)	38 (97.4)	
Cardiac disease			
Yes	3 (15.8)	4 (10.3)	0.67
No	16 (84.2)	35 (89.7)	
Respiratory disease			
Yes	0	3 (7.7)	0.54
No	19 (100.0)	36 (92.3)	
Gastrointestinal disease			
Yes	0	1 (2.6)	1.00
No	19 (100.0)	38 (97.4)	
Medication with muscle wasting consequence			
Yes	0	0	–
No	19 (100.0)	39 (100.0)	

## Results

Fifty-eight patients were recruited with 12 males and 46 females. Nineteen (32.8%) patients had sarcopenia at baseline. The mean age of sarcopenic subjects and non-sarcopenic subjects was comparable (67.89 ± 7.07 vs. 67.92 ± 6.85; *p* = 0.99) but sarcopenic subjects had a lower BMI (25.64 ± 2.64 vs. 28.57 ± 4.04; *p* = 0.01). Background medical comorbidities were comparable between the two groups. Patients with sarcopenia stayed slightly longer in the hospital after surgery despite not being statistically different from patients without sarcopenia (8.11 vs. 7.39 days, *p* = 0.61). The demographic characteristics of the patients are summarised in Table 1.

### Primary outcome measures

**Fig. 1:**
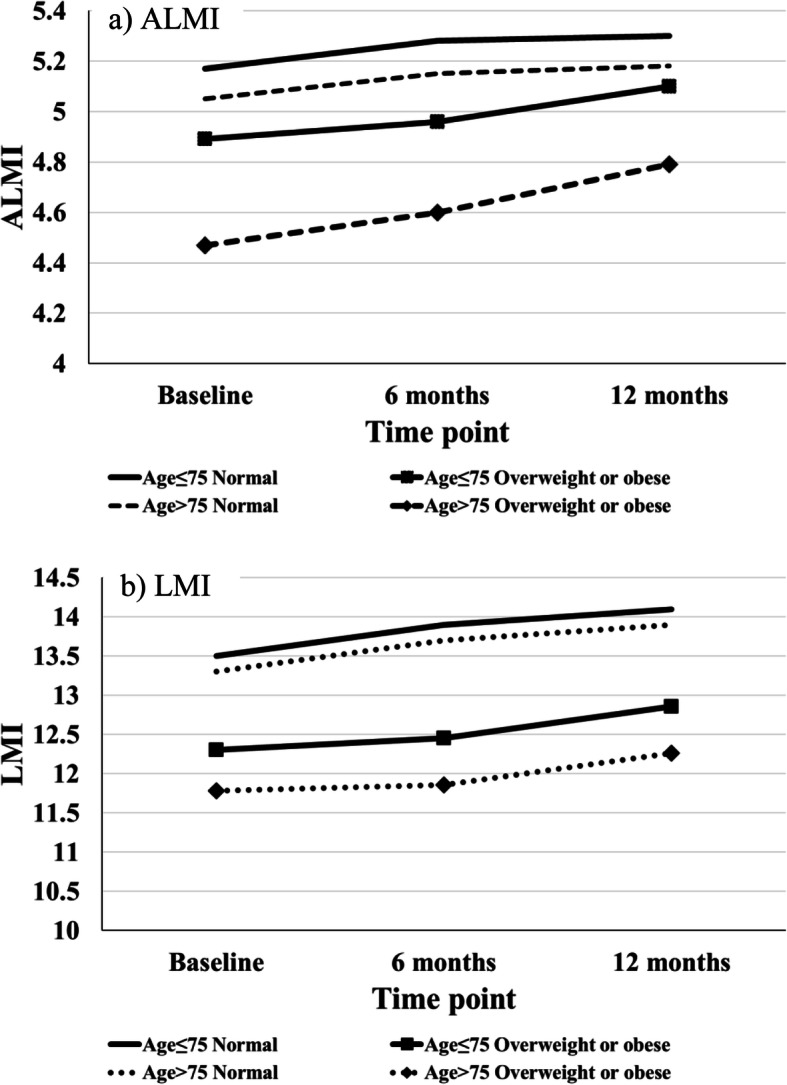
Muscle Mass changes after TKA: Longitudinal changes of **a**) Appendage Lean Mass Index (ALMI) and **b**) Lean Mass Index (LMI) scores in sarcopenia patients of different age groups and BMI categories

Improvement trends in muscle mass in both sarcopenic and non-sarcopenic patients were observed at 12 months (LMI in sarcopenic: 12.93 ± 1.27 (baseline) to 13.27 ± 1.3 (12 months), *p* = 0.14; LMI in non-sarcopenic: 14.96 ± 1.83 (baseline) to 15.42 ± 2.01 (12 months), *p* = 0.06)) (Table 2). After controlling for possible confounders, it was found that sarcopenic females that were overweight or obese had statistically significant improvements in both ALMI ([age ≤ 75, female, overweight or obese]: 4.89 (Baseline) vs. 4.96 (6 months) vs. 5.10 (12 months); *p* = 0.04) and [Age > 75, female, overweight or obese]: 4.47 vs. 4.60 vs. 4.79; *p* = 0.05) and LMI ([age ≤ 75, female, overweight or obese]: 12.30 vs. 12.45 vs. 12.86; *p* = 0.04 and [age > 75, female, overweight or obese]: 11.78 vs. 11.86 vs. 12.26; *p* = 0.04) after total knee arthroplasty (Table 3) (Fig. 1a and b). Nevertheless, despite the increase in muscle mass after TKA, both the ALMI and LMI in sarcopenic subjects remained lower than non-sarcopenic subjects at 12 months with statistical significance ([ALMI] at baseline: 5.26 (sarcopenia = yes) vs. 6.11 (sarcopenia = no); *p* < 0.01; 6 months: 5.22 vs. 6.15; *p* = 0.02; 12 months: 5.38 vs. 6.28; *p* < 0.01) ([LMI] at baseline: 13.10 (sarcopenia = yes) vs.14.96 (sarcopenia = no); *p* < 0.01; 6 months: 12.99 vs. 15.06; *p* = 0.01; 12 months: 13.39 vs. 15.42; *p* < 0.01) (Table 4).

**Table 2:** Longitudinal comparisons of Appendage Lean Mass Index and Lean Mass Index in patients with or without sarcopenia

DXA	Sarcopenia	Baseline	Time point		*P* value^a^
			6 months	12 months	
ALMI	Yes	5.26 ± 0.82	5.22 ± 0.81	5.38 ± 0.85	0.09
LMI		13.10 ± 1.44	12.99 ± 1.21	13.39 ± 1.38	0.14
ALMI	No	6.11 ± 0.89	6.15 ± 1.01	6.28 ± 1.03	0.07
LMI		14.96 ± 1.83	15.06 ± 1.97	15.42 ± 2.01	0.06

*ALMI* appendage lean mass index, Appendage lean mass/height^2^; Lean Mass Index, *LMI* total lean mass/height^2^

^a^ *post-hoc* Bonferroni log-rank test

**Table 3:** Longitudinal comparisons of Appendage Lean Mass Index and Lean Mass Index in patients with and without sarcopenia

Sarcopenia	Sex	Age	BMI	DXA variables	Time points			*P* value^b^
					Baseline	6 months	12 months	
Yes	Male	≤75	Normal	ALMI	6.34 ± 0.35	6.56 ± 0.29	6.37 ± 0.34	0.44
			Overweight or obese	ALMI	6.03 ± 1.46	6.51 ± 1.67	6.79 ± 0.21	0.40
		> 75	Normal	ALMI	5.07 ± 0.09	5.38 ± 0.11	5.28 ± 0.15	0.42
			Overweight or obese	ALMI	5.35 ± 0.47	5.23 ± 0.17	5.21 ± 0.14	0.48
		≤75	Normal	LMI	14.45 ± 0.64	14.60 ± 0.35	14.65 ± 0.07	0.45
			Overweight or obese	LMI	14.50 ± 2.52	14.90 ± 1.58	15.50 ± 0.57	0.44
		> 75	Normal	LMI	12.22 ± 1.19	12.34 ± 1.69	12.54 ± 0.68	0.46
			Overweight or obese	LMI	13.39 ± 0.87	14.09 ± 0.62	13.76 ± 0.95	0.45
	Female	≤75	Normal	ALMI	5.17 ± 0.17	5.28 ± 0.52	5.30 ± 0.15	0.31
			Overweight or obese	ALMI	4.89 ± 0.15	4.96 ± 0.15	5.10 ± 0.38	0.04^a^
		> 75	Normal	ALMI	5.05 ± 0.07	5.12 ± 0.10	5.18 ± 0.10	0.34
			Overweight or obese	ALMI	4.47 ± 0.43	4.60 ± 0.41	4.79 ± 0.52	0.05^a^
		≤75	Normal	LMI	13.50 ± 0.28	13.90 ± 0.42	14.10 ± 0.28	0.34
			Overweight or obese	LMI	12.30 ± 1.01	12.45 ± 1.35	12.86 ± 0.21	0.04^a^
		> 75	Normal	LMI	13.30 ± 0.27	13.70 ± 0.26	13.90 ± 0.72	0.36
			Overweight or obese	LMI	11.78 ± 0.77	11.86 ± 1.13	12.26 ± 0.88	0.04^a^
No	Male	≤75	Normal	ALMI	6.44 ± 0.57	6.21 ± 0.22	6.54 ± 0.43	0.39
			Overweight or obese	ALMI	7.69 ± 0.55	8.04 ± 0.64	7.95 ± 0.86	0.39
		> 75	Normal	ALMI	7.37 ± 0.37	7.54 ± 0.69	7.46 ± 0.12	0.38
			Overweight or obese	ALMI	7.89 ± 0.50	8.21 ± 0.66	7.94 ± 0.74	0.42
		≤75	Normal	LMI	14.70 ± 1.44	14.25 ± 0.49	14.70 ± 0.85	0.45
			Overweight or obese	LMI	17.80 ± 1.29	18.83 ± 1.05	18.45 ± 1.93	0.33
		> 75	Normal	LMI	16.40 ± 1.59	16.90 ± 0.38	16.65 ± 0.35	0.31
			Overweight or obese	LMI	17.98 ± 1.41	18.54 ± 1.02	18.39 ± 1.84	0.34
	Female	≤75	Normal	ALMI	4.85 ± 0.25	4.74 ± 0.23	4.72 ± 0.22	0.41
			Overweight or obese	ALMI	6.05 ± 0.53	5.92 ± 0.66	6.06 ± 0.56	0.36
		> 75	Normal	ALMI	4.59 ± 0.05	4.28 ± 0.14	4.44 ± 0.22	0.49
			Overweight or obese	ALMI	5.90 ± 0.34	5.95 ± 0.57	6.27 ± 0.53	0.27
		≤75	Normal	LMI	12.17 ± 0.51	12.00 ± 0.28	12.25 ± 0.49	0.43
			Overweight or obese	LMI	14.90 ± 1.10	14.88 ± 1.38	15.11 ± 1.30	0.40
		> 75	Normal	LMI	12.00 ± 1.34	11.40 ± 1.36	11.70 ± 0.42	0.48
			Overweight or obese	LMI	14.80 ± 1.70	14.15 ± 0.92	15.25 ± 0.99	0.34

*ALMI* appendage lean mass index, Appendage lean mass/Height^2^, *LMI* lean mass index: Total lean mass/Height^2^

^a^ Statistical significance using ANOVA

^b^ *post-hoc* Bonferroni log-rank test

**Table 4:** Cross-sectional comparisons of Appendage Lean Mass Index and Lean Mass Index between patients with and without sarcopenia in the 3 time points

DXA scores	Time point	Sarcopenia		*P* value^a^
		Yes	No	
ALMI	Baseline	5.26 ± 0.82	6.11 ± 0.89	< 0.01
	6 months	5.22 ± 0.81	6.15 ± 1.01	0.02
	12 months	5.38 ± 0.85	6.28 ± 1.03	< 0.01
LMI	Baseline	13.10 ± 1.44	14.96 ± 1.83	< 0.01
	6 months	12.99 ± 1.21	15.06 ± 1.97	0.01
	12 months	13.39 ± 1.38	15.42 ± 2.01	< 0.01

*ALMI* appendage lean mass index, Appendage lean mass/height^2^; *LMI* lean mass index: total lean mass/height^2^

^a^ *post-hoc* Bonferroni log-rank test

### Secondary outcome measures

**Fig. 2:**
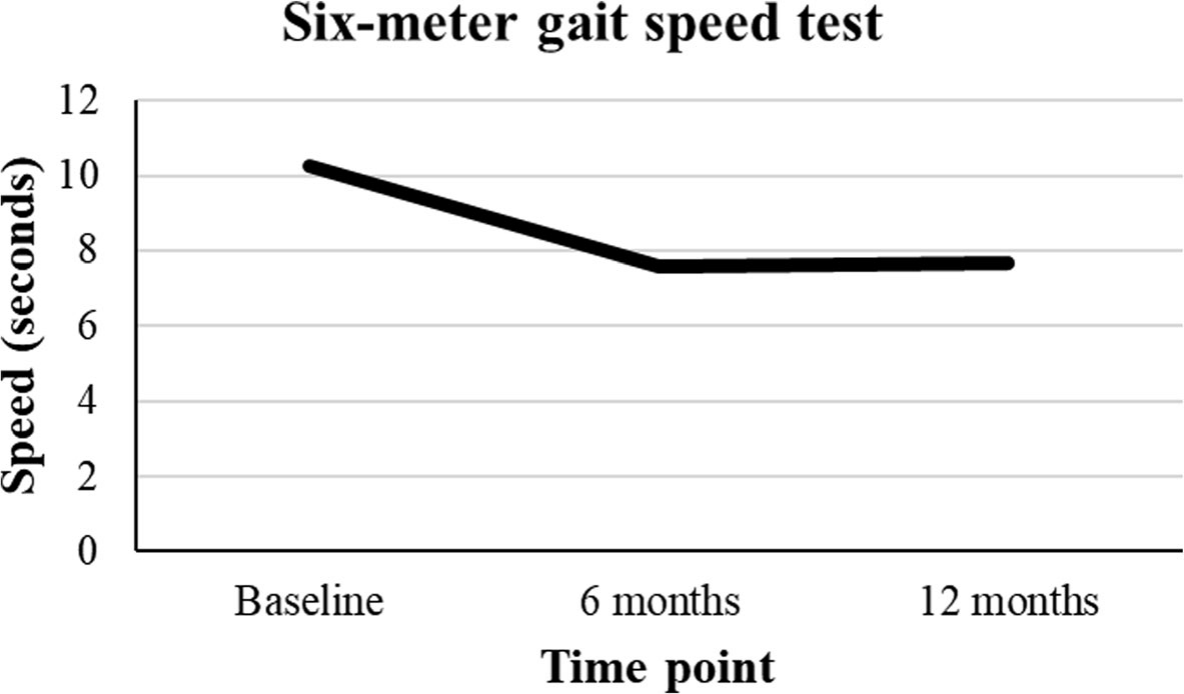
Muscle function changes after TKA: Six-meter gait speed test across the time points

**Fig. 3:**
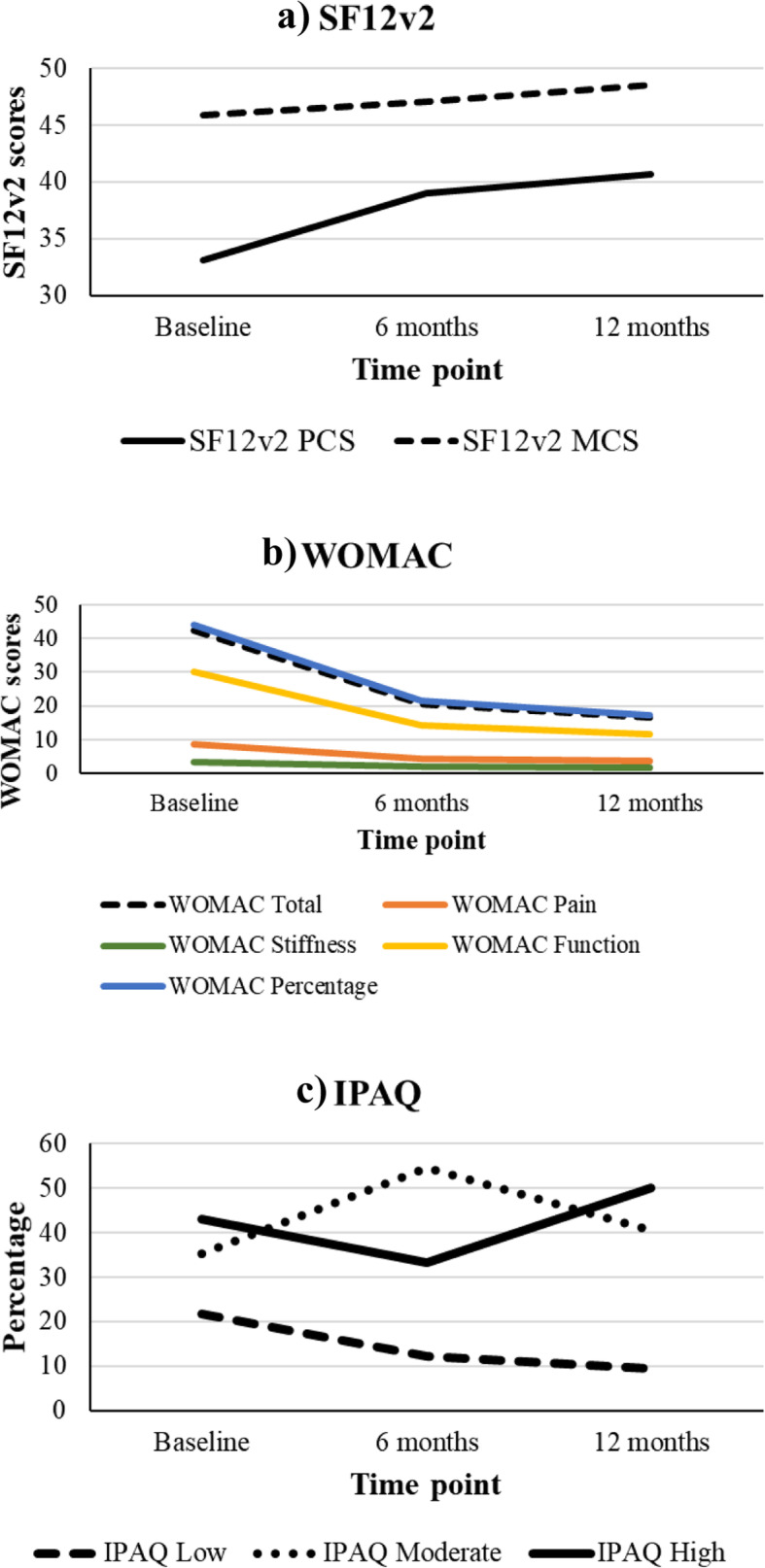
Quality of Life changes after TKA: Longitudinal changes of **a**) SF12v2, **b**) WOMAC, and **c**) IPAQ showing a gradual improvement in physical function and decreased pain after total knee arthroplasty

Statistically significant improvements in walking speed in both sarcopenic and non-sarcopenic patients were found as evidenced by reduced time in the 6-m gait speed test (10.24 ± 5.35 (baseline) to 7.69 ± 2.68 (12 months), *p* < 0.01) (Table 5) (Fig. 2). There were statistically significant improvements in operated knee extension muscle strength (12.56 vs. 15.53, *p* < 0.01) and flexion muscle strength (5.34 vs. 6.53, *p* = 0.03) in both sarcopenic and non-sarcopenic patients after TKA (Table 5). There was no change in handgrip power before and after TKA and subsequent follow-up (19.31 (baseline) vs. 18.98 (6 months) vs. 19.36 (12 months); *p* = 0.97) (Table 5). Patient outcome measures kept improving in terms of the WOMAC pain domain (baseline vs. 6 months vs. 12 months = 8.67 vs. 4.32 vs. 3.73, *p* < 0.01), stiffness domain (3.48 vs. 2.03 vs. 1.77, *p* < 0.01) and function domain (30.12 vs. 14.26 vs. 11.69, *p* < 0.01). The physical component score of the SF12v2 also echoed the improvement (33.06 vs. 38.96 vs. 40.67, *p* < 0.01). In conjunction with this trend, the percentage distribution of IPAQ ratings showed increased engagement of high-intensity activities (Fig. 3a and b).

**Table 5:** Longitudinal comparisons of SF12v2, WOMAC, IPAQ, and Functional Assessments of all patients

Questionnaires and Functional Assessments	Baseline	Time point		*P* value^a^
		6 months	12 months	
SF12v2				
PCS	33.06 ± 8.55	38.96 ± 8.01	40.67 ± 7.93	< 0.01
MCS	45.87 ± 9.70	47.04 ± 10.53	48.50 ± 10.19	0.46
WOMAC				
Total	42.27 ± 15.98	20.65 ± 15.24	16.65 ± 18.13	< 0.01
Pain	8.67 ± 3.51	4.32 ± 3.20	3.73 ± 4.62	< 0.01
Stiffness	3.48 ± 1.81	2.03 ± 1.70	1.77 ± 2.07	< 0.01
Function	30.12 ± 11.96	14.26 ± 11.43	11.69 ± 12.86	< 0.01
Percentage	44.03 ± 16.64	21.51 ± 15.87	17.35 ± 18.88	< 0.01
IPAQ				
Low	11 (21.6)	4 (12.1)	4 (9.5)	0.24
Moderate	18 (35.3)	18 (54.5)	17 (40.5)	
High	22 (43.1)	11 (33.3)	21 (50.0)	
Knee flexion/extension muscle strength				
Operated knee extension	12.56 ± 6.23	10.80 ± 4.99	15.53 ± 7.98	< 0.01
Operated knee flexion	5.34 ± 2.92	4.61 ± 2.49	6.53 ± 3.85	0.03
Non-operated knee extension	14.19 ± 7.61	14.07 ± 7.80	15.18 ± 8.36	0.79
Non-operated knee flexion	5.55 ± 3.13	5.49 ± 2.48	6.74 ± 3.94	0.15
Handgrip muscle strength	19.31 ± 5.92	18.98 ± 6.37	19.36 ± 7.66	0.97
Six-meter gait speed test	10.24 ± 5.35	7.56 ± 2.14	7.69 ± 2.68	< 0.01

*PCS* physical component score, *MCS* mental component score, *IPAQ* International Physical Activity Questionnaires

^a^ *post-hoc* Bonferroni log-rank test

### Adverse events

No adverse events were noted during this study.

## Discussion

Our study illustrates a high prevalence of sarcopenia among patients with end-stage OA of the knee. There were 58 patients at baseline, of which 19 (32.8%) had sarcopenia and 39 (67.2%) did not. The prevalence of sarcopenia in Asia ranges from 6.7 to 18.6% in older men and 0.1 to 23.6% in older women, according to various reports from Japan, Taiwan, Hong Kong and Korea [34–37]. However, it has also been found that the prevalence of sarcopenia among community-dwelling older patients with OA is near three times that of those without OA, which possibly explains the relatively high prevalence of sarcopenia among our OA subjects [38].

This study demonstrated that TKA can benefit patients with severe knee OA with or without co-existing sarcopenia by improving knee function and symptoms, in turn enhancing their lower limb muscle strength, gait speed and potentially lean muscle mass. It is important to note that deficits in gait speed, muscle strength and lean muscle mass are the core components that define sarcopenia. According to the latest review in *The Lancet* on sarcopenia, physical activity is regarded as the primary treatment for sarcopenia, as there are currently no specific drugs approved for the treatment of sarcopenia [39]. Our study illustrates the importance of identifying sarcopenic patients with concomitant joint disease and managing these patients accordingly to facilitate physical activity, which may in turn benefit their concomitant sarcopenia. At the end of this study, five sarcopenic patients at baseline turned non-sarcopenic, leading to a total of 44 patients without sarcopenia (75.9%). However, our results also show that knee arthroplasty alone cannot allow sarcopenic subjects to pick up the overall difference in average lean muscle mass compared to non-sarcopenic subjects. This highlights the importance of managing sarcopenia through a multimodal approach, for example a combination of high protein diet, knee arthroplasty and a supervised exercise program which by then should be more effective as the physical limitation due to knee OA has been alleviated. In our study, the patients only received standard physiotherapy designed for rehabilitation after of knee arthroplasty surgery to improve knee range and walking ability; this did not target building skeletal muscle strength and mass as would be found in a resistance exercise program for sarcopenia. Having said that, some of these OA patients were older patients with low motivation and were content with pain-free knees without further interest in participating in further sarcopenia muscle strengthening exercises. As such, some passive physical interventions or “exercise mimetics” like neuromuscular electrical stimulation or whole-body vibration may be considered for older patients [40]. In fact, whole-body vibration has been shown to increase knee extensor strength and decrease lower leg swelling after TKA and is thus worth further investigation for a combined effect on sarcopenia [41].

To the best of our knowledge, our study is the first one to observe the status of sarcopenia after TKA longitudinally, monitoring changes in muscle strength, muscle mass and gait speed over time. Previous studies have focused on pre-operative sarcopenia as a risk factor for poor surgical outcome. For example, sarcopenia has been identified as a risk factor for morbidity and mortality in colorectal surgery and gastric cancer surgery, and also as a risk factor for prosthetic infection after joint arthroplasty [26, 42, 43]. In our study, no increase in infection rate nor other complications were found; nevertheless, the timeframe for late infection and late complications was beyond this study period. One important difference between the current study and the previous research on sarcopenia with surgery is that those surgeries mainly induced a catabolic status in the patients while knee arthroplasty induces catabolism in the early phase followed by anabolism due to the patient regaining their mobility and ability to exercise. This phenomenon may potentially explain the improvement in lean mass in overweight or obese sarcopenic female subjects in our study, as they lost fat and weight during the initial catabolism after arthroplasty and built up muscle during their subsequent rehabilitation, made possible by better walking ability and less body weight hindering movement [19, 38, 44]. However, the finding is limited by the small scale of our study and further studies with larger sample sizes are warranted to validate this relationship.

There are certain limitations to our study. Firstly, all patients received standard physiotherapy in the early phase for post-op rehabilitation. Afterwards, we did not restrict or prescribe further exercise to patients and they may have engaged in variable degrees of exercise. This could have contributed to the variable improvement in muscle mass among our patients. Nevertheless, we found in general that they engaged in more exercise as reflected by improvement in the WOMAC function domain, physical component score of the SF12v2 and higher percentage distributions of IPAQ ratings of high-intensity activities (Fig. 3). Similarly, although we encouraged our patients to have high protein intake according to a dietitian’s advice, we could not control the exact patient diet at home; those with a relatively higher protein diet may have had better muscle mass building than their counterparts [39]. Besides, as there have been no previous studies looking into this topic, the sample size in this study was based on our pilot data and was small. A larger replicating study may help confirm the change in muscle mass over time, for which we showed only a trend for improvement without statistical significance in the overall sarcopenic group. Another limitation is that our study examined sarcopenia using the Asian Working Group for Sarcopenia (AWGS) algorithm and therefore the results may not be applicable to other ethnicities, for example to Caucasians in which sarcopenia is diagnosed by the consensus definition of the European Working Group on Sarcopenia in Older People (EWGSOP2) [45]. Future population-based studies on other ethnicities with different lifestyles may provide a more comprehensive understanding of the interrelationship between TKA and sarcopenia.

## Conclusions

To conclude, our study showed that sarcopenia among patients with end-stage OA of the knee is not uncommon. Total knee arthroplasty can provide significant improvement in pain, stiffness and function in sarcopenic OA patients. Domains of sarcopenia like muscle strength and gait speed showed improvement after TKA. Further studies with larger sample sizes and different ethnicities can help ascertain the impact of TKA on sarcopenic OA subjects.

## Data Availability

The datasets used and/or analysed during the current study are available from the corresponding author on reasonable request.

## Abbreviations

ALMI: Appendage lean mass index
ANOVA: Analysis of variance
BMI: Body mass index
DXA: Dual-energy X-ray absorptiometry
IPAQ: International Physical Activity Questionnaires
LMI: Lean mass index
MCS: Mental component score
OA: Osteoarthritis
PCS: Physical component score
QOL: Quality of life
SD: Standard deviation
SF12v2: Short Form 12 Version 2
TKA: Total Knee Arthroplasty
WOMAC: Western Ontario and McMaster Universities Arthritis Index
